# Techniques to cope with missing data in host–pathogen protein interaction prediction

**DOI:** 10.1093/bioinformatics/bts375

**Published:** 2012-09-03

**Authors:** Meghana Kshirsagar, Jaime Carbonell, Judith Klein-Seetharaman

**Affiliations:** ^1^School of Computer Science, Carnegie Mellon University 15213; ^2^Department of Structural Biology, University of Pittsburgh, School of Medicine, Pittsburgh 15261, USA; ^3^Forschungszentrum Jülich, Institute of Complex Systems (ICS-5), Jülich 52425, Germany

## Abstract

**Motivation:** Approaches that use supervised machine learning techniques for protein–protein interaction (PPI) prediction typically use features obtained by integrating several sources of data. Often certain attributes of the data are not available, resulting in missing values. In particular, our host–pathogen PPI datasets have a large fraction, in the range of 58–85% of missing values, which makes it challenging to apply machine learning algorithms.

**Results:** We show that specialized techniques for missing value imputation can improve the performance of the models significantly. We use cross species information in combination with machine learning techniques like Group lasso with *ℓ*_1_/*ℓ*_2_ regularization. We demonstrate the benefits of our approach on two PPI prediction problems. In our first example of *Salmonella*–human PPI prediction, we are able to obtain high prediction accuracies with 77.6% precision and 84% recall. Comparison with various other techniques shows an improvement of 9 in F1 score over the next best technique. We also apply our method to *Yersinia*–human PPI prediction successfully, demonstrating the generality of our approach.

**Availability:** Predicted interactions, datasets, features are available at: http://www.cs.cmu.edu/~mkshirsa/eccb2012_paper46.html.

**Contact:** judithks@cs.cmu.edu

**Supplementary Information:** Supplementary data are available at Bioinformatics online.

## 1 INTRODUCTION

Identification of protein–protein interactions (PPIs) between a pathogen and its hosts will not only give us insights into the molecular mechanisms underlying pathogenisis but also provide us with potential targets aiding in the development of pathogen-specific therapeutics. Computational methods complement experimental methods in studying PPIs by predicting highly probable interactions, which are used by experimentalists to filter out unlikely interactions from the prohibitively large set of potential interactions that need to be tested. In particular, supervised machine learning methods use the existing known interactions as training data and formulate the interaction prediction problem in a classification setting, with target classes: ‘interacting’ or ‘non-interacting’. Features are derived on the data by integrating various attributes of proteins, which include protein sequences from Uniprot (UniProt Consortium, 2011), protein family from Pfam (Finn *et al.*, 2010), protein structure from PDB, gene ontology (GO) from the GO database (Ashburner *et al.*, 2000), gene expression from GEO (Barrett *et al.*, 2011), interactions between protein families from iPfam (Finn *et al.*, 2005), protein domain interactions from 3DID (Stein *et al.*, 2011), to name a few.

Although these attributes are available for some proteins, many of them remain unknown for various reasons: they are a part of ongoing experimental studies, difficulty of experimentation due to the nature of the protein, lack of efficient high-throughput techniques, limited pace of manual curation from published literature, etc. Such unavailable information results in missing values in the dataset. Missing values make the application of any machine learning algorithm and data analysis technique difficult, as most approaches were designed to work on complete or nearly complete data. Imputation of missing values involves exploiting available information about the data in order to best estimate the missing entries. Thus, the choice of an appropriate missing value imputation method is very important in order to build good predictive models.

Here, we develop methods to deal with missing values for host–pathogen PPI prediction. Prior work on PPI prediction within the supervised learning framework deals with missing values in one of the following ways. (Mohamed *et al.*, 2010) eliminate all data with missing values. (Qi *et al.*, 2005) and (Singh *et al.*, 2006) use a Random Forest (RF) classifier-induced imputation originally introduced by (Breiman, 2001), which uses mean values of features in combination with proximity to other instances. The abstaining variant of Adaboost originally introduced by (Schapire and Singer, 1999) has been applied by (Smeraldi *et al.*, 2010). The work by (Qi *et al.*, 2007) adds one extra binary ‘indicator’ feature for every existing feature to indicate its availability. (Dyer *et al.*, 2011) limit the data sources used to generate features so as to exclude the possibility of any missing values.

As the main application of our techniques, we consider *Salmonella*–human PPI prediction, which is an important step towards developing our understanding of Salmonello-sis, a disease that causes millions of infections and thousands of deaths every year world-wide (Graham, 2010). The bacterium *Salmonella* has 4533 protein sequences in the reference proteome set in Uniprot database, known molecular functions for 1058 proteins in GO, protein structures for 592 proteins in PDB and protein family information for 2978 proteins in Pfam database. Approaches that construct features integrating data from these databases will thus have many missing values since not all attributes are available for all proteins across all databases. In the example dataset of *Salmonella*–human PPI that has 62 known interactions, with our feature set we find that ≈58% of the interactions have at least one feature with missing values.

To cope with missing values, we can apply standard statistical imputation methods or develop application-specific methods that take advantage of the particular properties of the data being imputed. Our work falls in the second category and is novel in its use of data from other related species, in addition to information from the species of interest itself. To transfer this information from other closely related species to the species of interest, we use protein sequence alignment to define a measure of similarity between the proteins from the two species. We use ideas from nearest-neighbour-based methods to combine such cross-species data. Our techniques have several benefits:

1. PPI datasets can have a very high missing value rate. Methods that use the limited available features to impute a large number of missing features are likely to produce very biased models. Our method uses cross-species data in addition to the available features thus reducing this bias.
2. We make no independence assumptions on the features. Previous work in imputation for PPI assumes that all features are independent of each other. However, this is not always true, e.g., gene expression data are a time series of expression measurements and features derived from such data cannot be considered independent.
3. We avoid explicitly estimating the density of very high dimensional features in order to impute the values.

The method we present is general and can be used whenever there is available data from a related distribution. The proposed use of abundantly available cross-species data is analogous to sampling the missing values of a feature from another related feature distribution. We demonstrate the generality of our approach by successfully extending our modelling efforts to predict PPIs in *Yersinia pestis*.

## 2 APPROACH

### 2.1 Problem setup

We describe the problem setup in the context of *Salmonella*–human PPI prediction; however, the ideas are similar for other host–pathogen PPI prediction tasks. We use a binary classification framework defining an ‘instance’ to be a pair of proteins *<p*_s_,*p*_h_*>*, where one protein is the pathogen protein ‘*p*_s_’ (i.e. *Salmonella*) and the other is the host protein ‘*p*_h_’ (i.e. human). Features are defined for every protein-pair using various properties of the individual proteins and combining them all into a single feature vector. Details of our feature-set are listed in Table 2. These features are used with discriminative classifiers like support vector machine (SVMs).

We used the list of 62 interacting protein-pairs reported in (Schleker *et al.*, 2012)—these data form the ‘interacting’or ‘positive’ class. Since there is no experimental evidence about proteins that do not interact, we construct the ‘non-interacting’ or ‘negative’ class using a technique commonly used in PPI prediction—using random pairs of proteins obtained by sampling the set of all possible *Salmonella*–human protein pairs. Our dataset had 3592 unique *Salmonella*^1^ and 24431 unique human proteins, resulting in ≈90 million protein pairs. The missing value analysis we present in Section 2.2 was done on a small random subset ‘*ℳ*’ of size 40 000 pairs that includes all interacting pairs. These data were used to decide on imputation strategy and not our training data—the details of training and test sets can be found in Section 3.

### 2.2 Mechanism of the missing values

Let 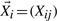, where *j* = 1,...*p*, be the feature vector corresponding to the *i*th protein pair. The *p* features can be considered random variables that have either been observed or missing. The seminal work by (Little and Rubin, 1987) identifies three broad categories of missing value mechanisms that are briefly listed below. Please refer to the original work for examples and details of each.

1. Missing completely at random (MCAR): if the probability that an observation *X_ij_* is missing is unrelated to its value or to the value of any other variable {*X_ik_* : *k* ≠ *j*}.
2. Missing at random (MAR): if the missingness of an observation *X_ij_* is independent of its value, given the value of some other observed variables in {*X_ik_* : *k* ≠ *j*}.
3. Missing not at random (MNAR): if the missingness depends both on the missing value itself and other observed variables.

The missingness mechanism in PPI datasets is MAR. Eliminating data that have any missing values, an approach called ‘listwise deletion’, has been followed in some prior work. However, it is appropriate only for data that is MCAR, otherwise it leads to biased estimates and models. Our claim of MAR arises from the observation that the absence of features for a given protein pair depends on how well studied the individual proteins are. An approximate measure of ‘well-studiedness’ for a protein can be obtained from UniprotKB, which defines an attribute called ‘protein existence’ that indicates the type of evidence supporting the existence of the protein. There are five categories of evidence: (1) evidence at protein level, (2) evidence at transcript level, (3) inferred from homology, (4) predicted and (5) ‘uncertain’. It is clear that proteins with status (1) or (2) can be considered to be well studied while the rest are not.

Let this ‘existence’ status of proteins be two additional observed features *E*_1_ and *E*_2_, then we can say that the missingness of other features in 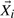 approximately depends on *E*_1_ and *E*_2_. It is independent of the actual value of every missing feature, thereby giving us an MAR mechanism. We observed that in the set ‘*ℳ*’ described in Section 2.1, the missingness of various features correlated well with *E*_1_ and *E*_2_ taking values of (3)–(5). Note how in this case, the removal of all data that have missing features will leave behind protein pairs that mostly involve well-studied proteins. A model built on such data will be undesirable as it will not glean any information about the less studied protein-pairs.

### 2.3 Imputation of missing values

Table 1 shows the percentage of missing values in our dataset, over all features and over individual feature types. The fraction of overall missing data is very high, about 58% for the positive class and 81% for the negative class. Furthermore, the missingness is highly skewed between the two classes, being significantly higher in the negative class data—this limits the choice of imputation techniques that can be used. Techniques like mean-value imputation can produce models that are biased towards predicting all data with the imputed features as ‘negative’. We also want to point out that high-dimensional features like the GO features are very difficult to impute using purely statistical modelling-based approaches that are very inefficient when the number of parameters to estimate is huge (≈180 million for the GO features).

**Table 1.** Percentage of examples with missing values

Features	% Missing in interacting pairs	% Missing in random pairs
Over all features	58.0	81.2
Gene Exp. GDS77	21.1	34.6
Gene Exp. GDS78	21.1	31.4
Gene Exp. GDS80	22.6	30.5
GO component	19.4	52.0
GO function	5.0	37.8
GO process	21.2	45.2

Shown over all features and also individually for the important features. Observations: the overall proportion of missing data is very high; the extent of missing values is highly skewed between the interacting and random pairs.

Considering these issues, we use a localized nearest-neighbour like approach that uses protein sequence similarity as the basis to compute the locality. The approach works in two phases: (i) cross-species information integration and (ii) modelling based imputation. The first component modifies the data source before feature computation by supplementing it with cross-species information. The latter uses modelling techniques to estimate feature values.

#### 2.3.1 Cross-species information integration

**Fig. 1.**
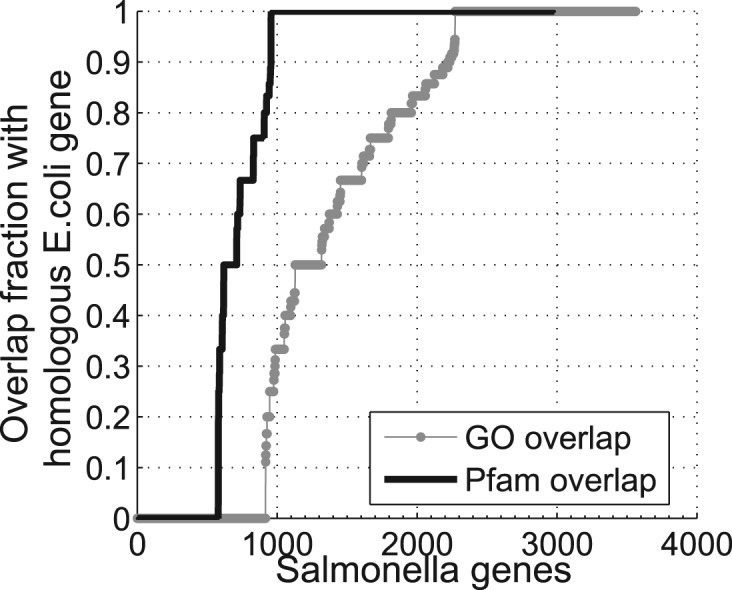
Overlap in GO terms for two closely related bacteria: *Salmonella* and *E.coli*. Each point on the curve represents the GO overlap of a *Salmonella* gene with the most homologous *E.coli* gene. Of all gene pairs, 68% show a high overlap in GO terms and 79% show a high overlap in Pfam

This technique relies on the observation that certain attributes of proteins like GO annotations and protein domains are similar for homologous proteins across various species. Whenever these properties are largely unavailable for any organism, we can exploit the vast amounts of equivalent information available in closely related organisms. For instance, the bacterium *Salmonella* is very similar to some other Gram-negative bacteria such as Escherichia coli, Francisella tularensis, Yersinia pestis, *Y.pestis*, etc. Some of these bacteria have been better studied and are likely to have more properties available than *Salmonella*. We illustrate the case of GO attributes in the context of two bacteria. The grey curve in Figure 1 shows the similarity in the GO terms^2^ between *Salmonella* and *E.coli* genes. The curve plots for every *Salmonella* gene (along *x*-axis), the overlap^3^ in the GO terms with the most homologous *E.coli* gene (along *y*-axis). Homologous^4^ gene pairs were obtained using BLAST alignment (*e*-value *<*0.01). This GO-overlap was computed only for *Salmonella* genes that had at least one GO annotation. Of these genes, 20% did not have any homologues in *E.coli* and are shown in the curve with the overlap value of 0. About 68% of the *Salmonella* genes have a GO-overlap of *>*50% with the corresponding homologous *E.coli* gene. We perform a similar analysis on protein family information (Pfam) for the two bacteria; the overlap is shown in Figure 1 (solid line).

**Imputing the GO features:** We used the above approach to impute GO annotations in both *Salmonella* and human proteins. For *Salmonella* proteins, we find proteins in all other bacteria which cross the BLAST *e*-value cutoff of 0.01 and use their GO annotations; whereas for human proteins, we use the GO annotations of aligning proteins from mouse and rat. The imputed value of a GO feature for a protein pair *<p*_s_,*p*_h_*>* where say the GO annotation of *p*_s_ is missing, uses the linear combination

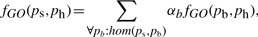

where *f_GO_*(*p*_b_,*p*_h_) computes the GO similarity feature between the proteins *p*_b_ and *p*_h_ using the method listed in Table 2, the function *hom*(.,.) checks if bacterial protein ‘*p*_b_’ is homologous to the *Salmonella* protein *p*_s_, and *α_b_* ∈[0,1] is the normalized similarity between *p*_s_ and *p*_b_. We use the BLAST alignment *e*-values to compute *α*_b_. Other cases where only *p*_h_, or both *p*_s_ and *p*_h_ are missing GO annotations are computed in an analogous manner.

**Table 2.** Feature set: summary of the various categories of features and the number of features in each category

Feature name (count)	Description
Gene ontology^a^ (≈177 million)	(i) Pairwise similarity: computed between GO terms of *p*_s_ and *p*_h_. Let *S* = set of all GO terms, *S*_p_ = set of GO terms for protein ‘*p*’. We set the entries of *S*× *S* corresponding to all pairs of GO terms from *S*_p_s__ × *S*_p_h__ to the similarity between the GO term pairs. Similarity between two individual GO-terms was computed using G-Sesame. (ii) Neighbour similarity: computed analogously using GO terms of *p*_s_ and binding partners of *p*_h_ in the human interactome. Total number of features from both categories = 2 |*S*|·|*S*|

Network-based (3)	Uses three graph properties of *p*_h_ in the human protein interaction network: (i) ‘degree’ = number of neighbours of *p*_h_; (ii) ‘clustering coefficient’ = ratio of edges present amongst neighbours of *p*_h_ to all possible edges between them; (iii) ‘centrality’ = fraction of shortest paths in the network that pass through *p*_h_

Gene expression (7)	Derived using the gene of the human protein *p*_h_. Uses three GEO datasets: GDS77, GDS78, GDS80 reporting differential gene expression of human genes infected by *Salmonella*, under seven different control conditions.

Conserved pathways (2)	We look for interactions *<p*_b_,*p*_h_2__> in other known bacteria–human PPI datasets, where bacterial protein *p*_b_ is a homologue of *p*_s_, and human proteins *p*_h_ and *p*_h_2__ share a pathway. Two such features were defined: (i) GP: aggregates bacteria–human PPI datasets across Gram-positive bacteria and (ii) GN: aggregated over Gram-negative bacteria. Let *P_c_* = number of human pathways shared by two human proteins; *Hom*(*p*_1_,*p*_2_) = 1 if the two proteins *p*_1_, *p*_2_ are homologous, 0 otherwise. Then, 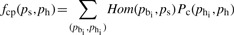

RNAi expression (2)	Uses human genes teemed as ‘hits’ by the RNAi screens published in (Misselwitz *et al.*, 2011). Defines two features: (i) pathway-enrichment: feature is ‘on’ if *p*_h_ belongs to a pathway statistically enriched by the RNAi hit genes. (ii) complex-enrichment: ‘on’ if *p*_h_ belongs to statistically enriched protein complexes. Enrichment analysis used the hypergeometric test with *p*-value cutoff of 0.01.

Interologues (1)	Number of protein-pairs from other species that are interologs of the given pair *<p*_s_,*p*_h_*>*.

Sequence *n*-grams (39 200)	Used the ‘conjoint triad model’ (Shen *et al.*, 2007) to get *n*-gram features on the protein sequence for *n*= 2, 3,4,5. The amino acid sequence is first converted to class sequence and *n*-grams are computed separately for *p*_s_ and *p*_h_ and then concatenated to give a single feature vector similar to Dyer *et al.* (2011) of size = 2(7^2^ +7^3^ +7^4^ + 7^5^).

Pfam interactions (1)	Counts the fraction of all possible interactions between the Pfam families of *p*_s_ and *p*_h_, that are listed as known interactions in the iPfam database (Finn *et al.*, 2005)

Domain interactions (1)	Similar to the above feature, computes the fraction of all possible domain–domain interactions between *p*_s_ and *p*_h_that are present in the domain interactions database 3DID (Stein *et al.*, 2011).

Protein sequence (2)	(i) Log of the *e*-value from the sequence alignment between proteins *p*_s_ and *p*_h_, computed using PSI-BLAST (Altschul *et al.*, 1997). (ii) Log of the *e*-value of the sequence alignment between *p*_s_ and *p*_h_'s binding partners in the human interactome. The maximum value is taken over all the binding partners.

*p*_h_ represents the human protein, and *p*_s_ represents the *Salmonella* protein in a given protein pair <*p*_s_,*p*_h_>.

^a^sparse features, i.e. only some of the millions of features are active in a single protein–pair.

The goal of our imputation method is to obtain a higher coverage over the missing data, at the same time maintaining good prediction accuracy. The *e*-value cutoff of 0.01 gave a good predictive performance. We also tried a lower threshold of 0.0001 and the results are discussed in Section 4.

**Imputing the gene expression features:** This is based on the premise that genes whose protein products are similar are likely to have a similar expression pattern. The imputed value for a protein pair *<p*_s_,*p*_h_*>* would be computed as

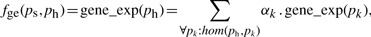

where ‘gene_exp()’ represents the expression of the gene encoding the given protein, *hom*(·, ·) and *α_k_* are defined as before.

#### 2.3.2 Modelling-based imputation

The above technique is able to transfer a large number of cross-species attributes, thereby reducing the fraction of missing data ranging from 58 to 80% to about 10–15% in both positive and negative class data. The proteins with the remaining missing attributes are either species-specific and do not have any homologues to transfer information from or have homologues which are also missing the attributes of interest. To deal with this much smaller fraction of remaining missing data, we use modelling-based imputation techniques.

**Modelling gene expression features:** Gene expression data are time-series data with several measurements spread across time or across different types of control experiments. Each expression value is not independent of the others, and it is important to model their distribution jointly. (Liew *et al.*, 2010) give a good review of missing value imputation for gene expression data. Our approach is different since it is related to PPIs. In addition to the gene expression data itself, we use protein sequence data to impute missing values. To use protein sequence data, we convert each sequence to a feature vector using the ‘conjoint triad’ concept from (Shen *et al.*, 2007). They partition the amino acids into seven classes based on their properties and replace every amino acid in the protein sequence by the appropriate class. We build *n*-gram features on this modified sequence for *n*= 2,3,4. This gives us 2793 features. Note how these features encode the protein sequence at a much coarser level; proteins with similar features are likely to have similar properties.

Given the observed gene expression data and sequence features for every protein, we model this imputation problem as a multivariate regression problem: *Y* = *XB*+*∈*. The covariates *X* ∈ℝ^*n* × *d*^ are the sequence features for *n* proteins and the response variables *Y* ∈ℝ*^n^*^×^*^k^* refer to *k* time-series gene expression values for the *n* proteins. We have 2793 sequence features that make *d* = 2793, and the set of all human proteins has *n*≈ 25 000 proteins. One of our gene expression datasets ‘GDS78’ downloaded from GEO (Barrett *et al.*, 2011), has three time-series measurements for each gene; thus *k* = 3. Since we want the response *Y* to be jointly dependent on a subset of the covariates *X*, we use the block regularization regression model from the work by (Obozinski *et al.*, 2008), also called ‘Group Lasso with *ℓ*_1_/*ℓ*_2_ regularization’. The sparsity constraints due to the *ℓ*_1_ norm in the formulation allow us to select a subset of the covariates (i.e. the sequence features) to regress upon. The *ℓ*_2_ norm allows for sharing of this covariate structure across the different response variables (i.e. sharing across the different time points). The details of this model, formulation and implementation are described inSubsection 3.1.

We also tried to regress each of the individual time points *Y_i_* separately using simple linear regression and squared error loss with lasso (*ℓ*_1_) regularization. However, in both these models the independence assumption between the response variables *Y* = {*Y_i_*} resulted in undesirable models. In order to illustrate that this imputation indeed improves the performance, we trained two models—one that discards examples with missing gene expression values, and the other that uses this regression-based imputation. We found an improvement of about 4% in F1.

## 3 METHODS

### 3.1 Imputation using group lasso with *ℓ*_1_/*ℓ*_2_ penalty

Group lasso with *ℓ*_1_/*ℓ*_2_ regularization (Obozinski *et al.*, 2008) is a way to perform high-dimensional multivariate linear regression in the scenario where the set of all response variables is to be regressed on the same set of covariates. Let *Y* ∈ℝ*^n^*^×^*^k^* be the matrix of real-valued observations, where each of the *n* observations comprises *k* responses. Let *X* ∈ℝ*^n×d^* be the design matrix with the set of covariates for each of the *n* observations. The multivariate linear regression model takes the form

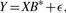

where B^*^∈ ℝ*^d^*^×^*^k^* is the regression coefficients matrix and *∈* ∈ℝ*^n^*^×^*^k^* is the noise matrix with zero mean. B^*^ has *d* coefficients corresponding to each of the *k* response variables *Y_i_*. For high-dimensional problems, a sparsity condition is assumed meaning that ‘most’ of the *d* covariates have zero weights across all the tasks. A natural way to solve for the coefficient matrix B^*^ is to formulate the regression as an optimization problem with the objective of minimizing the squared error, at the same time maintaining the sparsity of B^*^ as shown below:
(1)
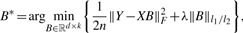

where *B*= (*β_ij_*) with 1≤*i*≤*d,* 1≤*j*≤*k*; rows representing the *d* covariates and columns representing the *k* responses and 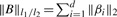 is the block *ℓ*_1_/*ℓ*_2_ norm; the norm ||·||*_F_* is the Frobenius norm of a matrix computed as the sum of squares of all individual elements of the matrix.

The *ℓ*_1_/*ℓ*_2_ norm has several nice properties. The *ℓ*_2_ norm couples together the columns of the coefficient matrix, as a consequence of which coefficients for a particular covariate jointly remain non-zero across all *k* responses. In our context this implies that, a particular sequence feature will be non-zero or active for the entire gene expression time series. Also, the *ℓ*_1_ norm decouples the rows of the coefficient matrix B^*^ and adds sparsity which results in some of the *d* rows getting selected as the important covariates and the remaining going to zero. This causes some of the sequence features getting selected as the important ones.

The optimization problem in Equation (1) is convex and is solved by using the dual augmented Lagrangian method for efficient sparse reconstruction, proposed by (Tomioka and Sugiyama, 2009). We use the implementation from (Puniyani *et al.*, 2010) and (Tomioka and Sugiyama, 2009). Parameters were tuned using 3-fold cross validation.

### 3.2 Dataset and training

We use the 62 *Salmonella*–human PPIs reported in (Schleker *et al.*, 2012) as the ‘positive class’ in our gold standard data. Call this set *P*. Let ‘*U*’ be the set of protein pairs obtained by pairing up all *Salmonella* proteins with all human proteins. Our proteins data described in Section 2.1 gave us |*U*| ≈90 million pairs. We sample some random pairs from the set *U*\*P*, to derive the set *N* that will be the negative class. The number of random pairs chosen to be the negative class is decided by what we expect the interaction ratio to be. We chose a ratio of 1:100 meaning that we expect 1 in every 100 random pairs of proteins to interact with each other, based on prior work in Dyer *et al.* (2010), Tastan *et al.* (2009). Our training data *D*= *P*∪*N* thus had 62 positives and 6200 negatives. To generate novel potential interactions given the gold-standard data, we apply the classifier model built on *D* to the rest of the protein pairs: (*U*\*D*) and report the positive pairs predicted by the classifier.

### 3.3 Classifiers for learning

In our techniques, we tried SVM (Boser *et al.*, 1992) and Logistic regression (LR) (Hastie *et al.*, 2009) with *ℓ*_1_ and *ℓ*_2_ regularization. A linear kernel was used for SVM experiments. Since our feature set is very high dimensional, the *ℓ*_1_ regularization-based methods were the most efficient. We found that LR with *ℓ*_1_ regularization (LR L1) had the best accuracy in all our experiments. This is explained by the fact that our feature set is very sparse and *ℓ*_1_ is known to learn sparse hypotheses well. We used the Liblinear implementation by (Fan *et al.*, 2008) for both SVM and LR.

### 3.4 Experimental setup

Our evaluation criteria were chosen considering the characteristics of our dataset: high class imbalance and small number of labelled examples. Instead of using accuracy that measures performance over both the classes, we use precision, recall and F1 computed on the positive class. Note how a classifier that labels all data as ‘negative’ will have a high accuracy but 0 recall.

#### 3.4.1 Bootstrap sampling experiments

Bootstrap sampling (Efron and Tibshirani, 1994) is used in statistical estimation when the available labelled examples are small in number. We perform 100 randomized bootstrap sampling experiments. For each experiment, we first make two random splits of 60 and 40% of the data, such that the class ratio of 1:100 is maintained in both. The training set is constructed using a bootstrap sample from the 60% split and the test data from the 40% split. Precision and recall are computed for each experiment and then averaged over the 100 experiments.

#### 3.4.2 Precision–recall curves

(PR) curves have been shown to give a more informative picture of an algorithm's performance than ROC curves in the case of high class imbalance (Davis *et al.*, 2006). The PR curve for every method was obtained by averaging over the 100 bootstrap sampling experiments.

#### 3.4.3 Leave-one-out cross-validation

This technique, also called ‘jack-knifing’ is known to give an almost unbiased estimate of the true error, as it uses most of the available labelled data. It is a cross-validation technique where one instance is left out in each iteration and used for testing. Say the gold standard dataset contains *P* positive examples. We perform *P* experiments leaving out one positive and 100 negative examples in each, while training the model on the remaining *P*−1 positives and (*P*−1)×100 negatives. This maintains the class ratio in all experiments at 1:100.

#### 3.4.4 Parameter tuning

To tune parameters, we perform a grid search over a range of possible parameter values and combinations of learning algorithms (SVM or LR) and type of regularization (*ℓ*_1_ or *ℓ*_2_). The parameters for SVM and LR are the tradeoff parameter *λ* and the class-specific loss parameters *C*_+_ and *C*_−_. We did a 5-fold nested cross validation (Varma and Simon, 2006), the results are reported in the Supplementary Material. The parameters that give best performance were chosen and fixed for all the random sampling and leave-one-out cross-validation (LOOCV) experiments. The same tuning technique was used for the alternate algorithms we compare against.

## 4 RESULTS

We evaluate our cross-species imputation technique by comparing it (i) with other imputation techniques and (ii) with other recent work in host–pathogen PPI prediction. Results are shown in Table 3 for the two scenarios. For the 100 bootstrap sampling experiments, along with precision and recall averaged across 100 runs, we also show the standard deviation ‘*σ* ’.

**Table 3.** First three columns show the performance of various techniques on precision, recall and F1 averaged over 100 bootstrap sampling experiments along with the standard deviation ‘*σ* ’. The last three columns show the precision, recall and F1 obtained using LOOCV. Bold entries correspond to highest accuracy achieved across the various methods. ‘RF’, Random Forest, ‘LR’, Logistic Regression; ‘L1’ refers to the *ℓ*_1_ regularizer. ^a^Did not finish running 100 bootstrap runs, so averaged over 30

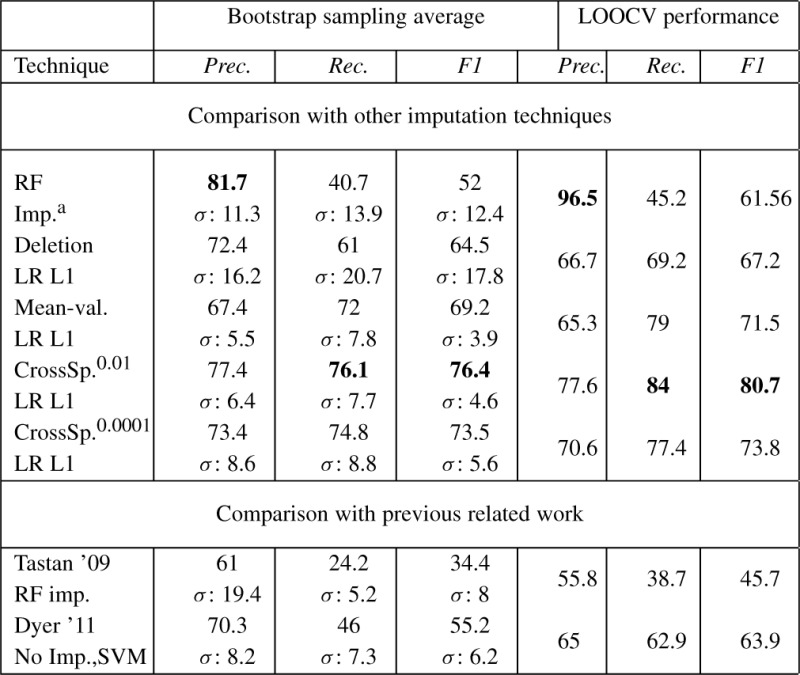

### 4.1 Comparison with other imputation methods

Both classifier-induced and data-driven imputation methods were applied on the feature-set described in Table 2. RF imputation is a classifier induced imputation, which works during the training phase of the classifier. The data driven imputation methods, namely mean value and cross-species, work before the classifier. Subsequently, we apply two classifiers on this imputed data: SVM and LR. For each classifier, we found that *ℓ*_1_ regularization outperforms *ℓ*_2_ regularization. We compared the following imputation approaches.

- RF imputation The implementation of RF by (Breiman and Cutler, 2004) imputes missing data by initializing each missing value to the mean of the feature and re-estimating values by building a forest and using nearest examples from the leaf nodes.
- Mean-value imputation Here, the average value of every feature is used to impute missing values. Feature averages obtained on the training data were used to impute both training and test data. Another related technique is ‘class-wise’or ‘multiple’mean-value imputation, where the averages are computed separately for the two classes. We tried this technique and the results did not improve over mean-value imputation.
- Cross species imputation This refers to our techniques described in Sections 2.3 and 3. We show results for two *e*-value thresholds: 0.01 and 0.0001 that define ‘homologous’ proteins in other species that will be used for imputation.
- Deletion imputation We simply discard all data with missing values. This leaves us with 26 interacting pairs; we pick a random set of 2600 random pairs to be the negative class, such that there are no features with missing values. We find that *ℓ*_1_ regularized LR, when applied on this reduced dataset performs better than SVMs or *ℓ*_2_ regularization.

In Table 3, we see that LR with *ℓ*_1_ regularization (LR L1) applied on the cross-species imputed data using a BLAST *e*-value threshold of 0.01 outperforms all other approaches, with an F1 score of 76.4. This is an improvement of about 7.2 points over the next best result of 69.2 achieved by LR L1 with mean-value imputation. Note that deletion imputation that uses a much smaller dataset has a very high variance (*σ* ≈18) in its performance. It is very sensitive to the dataset used and will produce a model that overfits the data, which is undesirable. The LOOCV results show that our technique has an F1 score of 80.7, which is 9 points better than the mean-value imputation technique. The LOOCV performance for all techniques is better than the bootstrap sampling experiments, since they use the maximum available training data. The improvement obtained by our method is evident from the percent increase in F1 that we achieve over the other imputation methods: 18.4% over deletion, 10.4% over mean-value and 46.9% over RF imputation. Permutation tests comparing our technique with all others show that the gains are statistically significant.

**Fig. 2.**
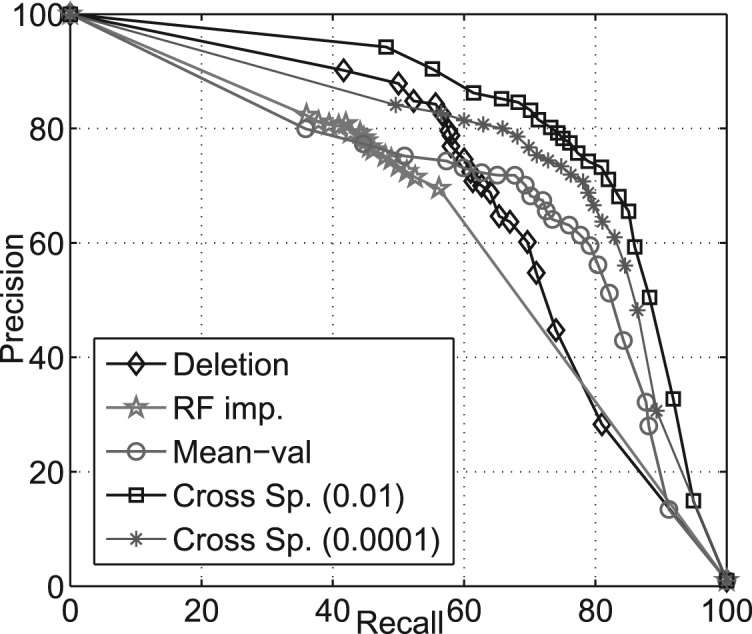
Precision–recall curve for various imputation techniques.

Figure 2 shows the PR curves for all the imputation techniques. RF imputation and deletion have good precision but suffer in recall; the opposite is true for Mean-val imputation. Cross species imputation however, does well on both precision and recall. All results are shown for positive to negative class skew of 1:100. We tried other class skews: 1:1, 1:50 and 1:150 and found that all techniques report higher accuracies for the lower class skews and the performance degrades gradually with increasing class skew. However, in all settings the ranking of relative performance remains the same: L1 LR with cross-species imputation performs the best, with the difference in performance being more on higher class skews. In the case of equally balanced class ratio (i.e. 1:1), all the curves come very close to each other.

## 4.2 Comparison with related work

Two methods originally proposed to address the HIV-human PPI prediction problem were compared here.

Tastan *et al*. 2009 used RF with the built-in classifier-induced feature imputation technique from (Breiman and Cutler, 2004). Dyer *et al*. 2011 and assumed there are no missing values and applied. SVMs with a linear kernel.

For both methods, we derived their features on our dataset and applied their techniques. The results in Table 3 show the superiority of our combination of feature set, classifier and imputation method over previous work on host–pathogen PPI prediction.

### 4.3 Results on *Yersinia*–human PPI dataset

We demonstrate the consistency of our techniques on* Yersinia pestis*–human PPI prediction. The dataset comes from high-throughput experiments (Dyer *et al.*, 2010) and has 4067 reported interactions. We build a negative class dataset using random pairs of proteins as described earlier, with a class ratio of 1:100. Features similar to the ones described in Table 2 were used. This PPI dataset also has several missing values as shown in Table 4. We apply the two best imputation techniques and show results for the 100 bootstrap-sampling experiments in Table 5. The overall accuracies are lower for this dataset as it comes from high-throughput experiments that tend to be noisy, and also because it has a larger proportion of missing values.

**Table 4.** Percentage of examples with missing values in the *Yersinia*–human PPI dataset, for all features and separately for two features

Type	% Miss in positive	% Miss in random
All features	83%	85%
Gene expression	15–27%	24–43%
GO	39–66%	40–54%

**Table 5.** Bootstrap sampling results on *Yersinia*–human PPI prediction comparing different imputation techniques

Method	Prec.	Rec.	F1
Mean Value	36.8	42.4	39.1
Cross species	44.6	42.8	43.7

## 5 CONCLUSION

We presented an imputation technique for dealing with missing values that arise in PPI prediction tasks. The improvements we obtain on both host–pathogen PPI datasets over prior approaches that use generic imputation methods clearly show the superiority of our method. We further observe that the importance of the imputed features in the learned classifiers is significant, implying that their imputation is important for prediction. Although our current work shows improvements in accuracy, we believe that the resulting models which are based on the MAR missingness assumption are less biased by the imputation technique as compared to the other commonly used methods. Characterizing and measuring such model bias as a consequence of imputation will be an interesting direction for future study, as it will make model selection more principled.

*Funding*: In part, Richard King Mellon Foundation, EraSysBio+ from the European Union and BMBF to SHIPREC, NIH (P50GM082251 and 2RO1LM007994-05), and NSF (CCF-1144281).

*Conflict of Interest*: none declared.

## Footnotes

^1^We consider strains Typhimurium, Typhi and Enteritidis

^2^Terms from the three ontologies: cellular component, molecular function and biological process were combined for this analysis.

^3^Overlap = intersection of the two sets of GO terms or Pfam ids.

^4^We use ‘homologue’ in a loose sense for sequences similar to each other.
